# Supplementary material for: LeishTec vaccination disrupts vertical transmission of Leishmania infantum
Source: PLoS Negl Trop Dis. 2025 Jul 30;19(7):e0013320. doi: 10.1371/journal.pntd.0013320 (PMC12321126; doi:10.1371/journal.pntd.0013320)
Supplement: Attachment — Submitted filename: Response to Reviewers.pdf [file pntd.0013320.s003.pdf]

**Response to Reviewers and Editor**

**Manuscript Title**: *LeishTec vaccination disrupts vertical transmission of Leishmania infantum.*

**Manuscript Number**: PNTD-D-24-01331
**Journal**: *PLOS Neglected Tropical Diseases*

We would like to thank the reviewers and editors for their thorough and insightful comments, which have helped us improve the quality and clarity of our manuscript. Below, we provide a detailed point-by-point response to each comment. All changes made in the manuscript are highlighted using track changes and highlighted in yellow.

**Additional Editor Comments:**

The study is highly significant as it investigates the use of a Leishmania vaccine in preventing the vertical transmission of L. infantum from infected mothers to their offspring. Based on my evaluation of the manuscript, I believe it requires major revisions before it can be accepted for publication.

Despite differing opinions among the reviewers (one recommending rejection, another suggesting major revisions, and a third suggesting minor revisions), I believe that undertaking major revisions is essential to improve the manuscript and make it suitable for publication.

As Reviewer 2 pointed out, PCR was performed on blood samples, which are not the most sensitive samples for this purpose. The authors should justify this choice and consider using more appropriate samples, such as bone marrow or spleen tissue. Additionally, it would be valuable to include an analysis of laboratory parameters, such as total protein and its fractions, creatinine levels, urinalysis, and UPC (urine protein-to-creatinine ratio).

As Reviewer 3 recommended, the manuscript should include an immunological analysis to explain how the vaccine blocks transmission to the puppies. Is this achieved through a humoral immune response, a cellular immune response, or both? A semi-quantitative immunological assessment of both the dams and the puppies should be conducted to address this question.

**Comment:**
Thank you for the feedback and opportunity to answer all questions raised in regards of our study. As described, this was a nested retrospective study built off of a vaccine clinical trial. More than 650 dogs were enrolled in the trial, and with those numbers a streamlined approach to assessment was necessary. We did not perform full clinicopathological assessment on this population which includes the dams for this present study over the 12-month vaccine trial, we focused on blood draw to determine change in *Leishmania* diagnostic status over time to establish vaccine efficacy. Similarly, in following diagnostic outcomes of the resultant pups, we were looking at surveillance for evidence of infection, and usually not clinical diagnosis of disease. Again, screening through blood draw was more efficient and effective for assessment of these large, cohoused populations vs. taking more invasive samples like bone marrow or lymph node aspirates or trying to obtain urine from a population that is freely available to urinate at any time and is unlikely to urinate on demand. In the rare instances where we were asked to perform further clinical workup, we have provided that data, but this is a subset of the overall population.

Bone marrow and spleen samples are more sensitive to detect genomic parasites equivalents by PCR. However, harvesting these tissues was not possible. Our cohort are valuable hunting dogs

so euthanasia was only possible when requested by the client. Spleen is very sensitive for diagnosing the presence of parasites however, due the high risk of lethality or complications with obtaining these samples, such procedure is abandoned for live animals and even forbidden for humans and canines by some endemic countries, such in Brazil. We did not have permission to perform more invasive diagnosis procedures, such bone marrow aspiration to perform RT-PCR in such samples, but only blood draw. In this revised work, to add rigor to our diagnosis of the pups, where possible we added adaptive immune response data and also LeishVet score based on biochemistry panel, complete blood count and clinical examination of these dogs. We agree that those parameters add immune mechanistic hypotheses and information regarding the challenges of diagnosing subclinical pups born to infected and vaccinated dams. We agree that subclinical dogs with very low parasitism are a diagnostic challenge, so further similar studies on different cohorts are needed to better understand this setting.

We are also submitting the main data set used for graphing figures in this manuscript as "Supporting Information".

## Methods

## Reviewer #1:

Reviewer #1: -Are the objectives of the study clearly articulated with a clear testable hypothesis stated? Yes
-Is the study design appropriate to address the stated objectives? Yes
-Is the population clearly described and appropriate for the hypothesis being tested? Yes
-Is the sample size sufficient to ensure adequate power to address the hypothesis being tested? No, but the authors acknowledge and address the sample size limitation.
**Response:** We appreciate the understanding on the feasibility of recruiting such a cohort.
-Were correct statistical analysis used to support conclusions? Yes
-Are there concerns about ethical or regulatory requirements being met? Yes

## Reviewer #2:

Reviewer #2: The objectives of the study is clearly articulated with a clear testable hypothesis stated but the study design is not appropriate for the hypothesis tested, as PCR was performed on a blood sample. This biological sample is not very sensitive for evaluating infection. It would be necessary to use a bone marrow or spleen sample. Furthermore, it would be important to analyze the laboratory dosages of total protein and fractions, creatine, urinalysis and UPC. Analyzing clinical signs alone is not sufficient to determine the presence of canine Leishmaniasis. Scientific studies demonstrate that there are patients without evident clinical signs with subclinical disease and moderate to high parasite load in the spleen and bone marrow.

**Response:** Thank you for your comments regarding test sensitivity and diagnosis in canine leishmaniosis. We agree that bone marrow and spleen samples are more sensitive to detect genomic parasites equivalents by PCR. However, collection of aspirates or tissue samples in the field was not a possibility. This hunting dog cohort requires sampling 50-100 dogs in a kennel setting. Because of the complications to splenic aspirates, such procedure is abandoned for live animals and even forbidden for humans and canines by endemic countries, such in Brazil. Additionally, we did not had permission to perform more invasive diagnosis procedures, such

bone marrow aspiration to perform RT-PCR in such samples, but only blood draw. In the studies of the pups where possible we have added LeishVet score information as we did perform smaller studies of these populations that allowed processing of biochemistry panel CBC and clinical examination of these dogs.

The population clearly described and appropriate for the hypothesis being tested? Yes.
Is the sample size sufficient to ensure adequate power to address the hypothesis being tested? Yes.
Were correct statistical analysis used to support conclusions? Needs to be reviewed.
**Response:** We appreciate the reviewer's suggestion we have added more clinical information of the pups to provide a clearer clinical picture.
Are there concerns about ethical or regulatory requirements being met? Yes, there are.

## Reviewer #3:

Reviewer #3: -Are the objectives of the study clearly articulated with a clear testable hypothesis stated? Yes.
-Is the study design appropriate to address the stated objectives? No, the number of dams were not sufficient for a strong and comprehensive statistical analysis.
**Response:** We thank the reviewer for this observation; as this was a nested retrospective study it was not possible or feasible to recruit such specific cohort. We agree that the goal of this publication is not to claim that these dogs had clinical illness, but instead a provocative finding that needs to be tested in larger outbred cohort.

-Is the population clearly described and appropriate for the hypothesis being tested? No, this is the major problem of the manuscript.
**Response:** We thank you for pointing out. We revised statements that could lead to an overinterpretation of data, claiming conclusions outside the scope of this study. To strength our main hypothesis: "*We hypothesized that dogs born to infected and vaccinated dams would be less likely to test diagnostically positive via L. infantum specific kqPCR or serology compared to dogs born to infected unvaccinated mothers.",* we also added other approaches to address the hypothesis with a broader methodology. We have included CD4+ T cell responses from peripheral blood and LeishVet score for a subset of pups from vaccinated and unvaccinated dams. We revised our language to avoid data over interpretation where audience could misinterpret to be more clear that we are not claiming lack of infection/sterile cure from vaccination in pups without diagnostic evidence of infection because we surely agree these dogs could be subclinical with very low parasite burdens, and they could manifest disease at a later time as demonstrated by Petersen's group. We hope the reviewer will appreciate our efforts to provide this additional clinicopathological and immunologic information.

-Were correct statistical analysis used to support conclusions? Yes. However, with a limited force.
-Are there concerns about ethical or regulatory requirements being met? Yes

## Results

## Reviewer #1:

Reviewer #1: -Does the analysis presented match the analysis plan? Yes
-Are the results clearly and completely presented? Yes
-Are the figures (Tables, Images) of sufficient quality for clarity? Yes

**Comment: We appreciate the reviewer's feedback.**

## Reviewer #2:

Reviewer #2: Does the analysis presented match the analysis plan? yes.
Are the results clearly and completely presented? No, needs to be reviewed.
Are the figures (Tables, Images) of sufficient quality for clarity? The flowchar and demographic data is well designed and clearly defines the analysis and losses between the groups. The graphs on clinical signs could be better detailed regarding the intensity of each clinical sign and which signs were most prevalent.The graphs on antibody quantification do not define the cutoff point nor does it define which symbol represents the offspring of each group. The survival curve of the dams shows that unvaccinated bitches had a longer survival rate. This information needs to be discussed at work.

**Response**: Thank you for this observation as this is an important issue. Our group and other colleagues often avoid expressing intensity for clinical signs, because is not consistent between clinical professionals, instead presence or absence is more consistent. Although we are plotting percent of positive diagnosed dogs in offspring, we are adding the cut off for serological assays, to make this clearer. We have plotted by litter, but this gets very messy and convoluted and sadly more confusing. We apologize that this is the case. In regards of the "survival curve" mentioned by reviewer 2, we want to address this carefully. This is not survival, but a "time to event" plot with the outcome being diagnostic positivity, so instead the interpretation is that unvaccinated bitches took longer to become overtly diagnostically positive; which is why they were bred. We reviewed the statements to avoid confusion. Thank you for pointing this out.

## Reviewer #3:

Reviewer #3: -Does the analysis presented match the analysis plan? No, due to the limited number of dams included in the study.
**Response:** We appreciate the feedback, and we also hope that reviewer 3 appreciates the changes made and mentioned above where we have tried to provide a better clinical picture where possible.
-Are the results clearly and completely presented? No, several aspects of the results should be more addressed in the results, such as the humoral response.
**Response:** We thank the reviewer for this observation. We address humoral responses via serology in figure 2. We have added adaptive responses, represented as ratio for CD4 T cell IFNg by CD4 T cell IL-10 secretion, in a kinetic fashion. Similarly, we added LeishVet score from a subset of dogs in the cohort. We agree that those parameters add mechanistic data to provide a better clinical and possible rationale for protection in dogs born to infected and vaccinated dams, however with the caveat that they may be asymptomatic and further similar studies on different cohorts are needed to address with more understanding.
-Are the figures (Tables, Images) of sufficient quality for clarity? Yes

# Conclusion

## Reviewer #1:

Reviewer #1: -Are the conclusions supported by the data presented? Yes
-Are the limitations of analysis clearly described? Yes
-Do the authors discuss how these data can be helpful to advance our understanding of the topic under

study? Yes
-Is public health relevance addressed? Yes

**Comment: We appreciate the reviewer's feedback.**

## Reviewer #2:

Reviewer #2: Are the conclusions supported by the data presented? No.
Are the limitations of analysis clearly described? Yes. The results do not adequately assess the presence of infection or disease.
Spleen or bone marrow PCR should have been used to quantify the parasite load.
Total proteins and fractions, urinalysis and UPC should have been measured, among other biochemical analyzes that could evaluate canine leishmaniasis.
The authors conclude that the vaccination of dams allowed uninfected puppies, but they did not perform PCR on biological samples with a sufficient degree of sensitivity to define the parasite load.
The objective of the study is valid and its design is well defined, but the diagnostic tools were not adequate. It is a risk to public health, suggested that vaccination was able to block vertical transmission based on the results obtained in this work.
I suggest that the authors look into the possibility of collecting spleen or bone marrow samples for evaluation of these patients. And biochemical tests for analysis and staging of canine leishmaniasis according to the guide line by Solano-Gallego et al.
The discussion about the survival curve of dams should also be carried out.

**Comment:** We appreciate the reviewer's suggestion and observations. Several of points raised here we addressed previously in our responses, such as our inability to collect spleen and bone marrow samples or aspriates, and we hope reviewer 2 understands our limitations. As mentioned above, to include and answer reviewer's 2 critiques and suggestions, we have added one more figure including adaptive responses to *L. infantum* antigen, ratio for CD4 T cell IFNg by CD4 T cell IL-10 secretion, in a kinetic fashion. We also added LeishVet score from some of the dogs in the cohort, as suggested by the reviewer. Such data was not included previously because our group could not investigate all samples for all time points, but the n and such limitations are described in the new method and figure legend for the new figure 3. We agree that the goal of this publication is not claiming lack of diagnosis as an absolute block of parasite transmission from mother to offspring, but a provocative finding where immunotherapy of infected dams disrupts such transmission. This could lead to a very low and undetected-subclinical parasitism. But the possibility of offspring clearing parasites needs to be tested in larger cohort with more variable genetic background, rather than just a specific breed. We are adding the above statements in our conclusion to avoid overstatements and overinterpretation of our findings by the audience of the study.

## Reviewer #3:

Reviewer #3: -Are the conclusions supported by the data presented? No, due to the insufficient number of dams included in the study.
-Are the limitations of analysis clearly described? Yes.
-Do the authors discuss how these data can be helpful to advance our understanding of the topic under study? YEs, but with a limited emphasis.
-Is public health relevance addressed? Yes, but with a limited emphasis.

**Response:** We thank the reviewer for this feedback, and we hope the reviewer understands the limitation of the sample size, discussed above in the methods section. We also hope the reviewer 3 appreciates the importance of the discussion that our study provokes on the possibility of a different approach to help to control canine leishmaniasis. Our group has as a goal for this publication to promote discussion on strategies that may be feasible to help to control vertical transmission of canine visceral leishmaniasis. But, as mentioned above, we agree that our publication is a spark on that direction, and broader studies need to be done in larger cohort with more variable genetic background or even in different endemic areas. Such statements are included in the conclusion section.

## Additional Changes Noted by the Authors

In addition to the reviewer comments, we also made the following improvements to enhance the clarity and rigor of the manuscript:

- Added Figure 3 to improve study data appreciation on offspring immunity against Leishmania parasites and LeishVet score of dogs, including respective section on methods and figure legend.
- Added 2 authors that provided resources and data for new figure 3.
- Added statement in conclusion to avoid overinterpretation of data.
- Added 2 more references on CanL immunity and LeishVet guideline, as suggested by reviewers.
- Added Author Summary in manuscript main body, instead of a separate file.

Please let us know if any further clarifications are needed. We are grateful for the opportunity to revise our manuscript and hope that the changes made have addressed the reviewers' concerns satisfactorily.

Sincerely,
**Diogo Garcia Valadares**
On behalf of all co-authors
**Cedars Sinai – Los Angeles**

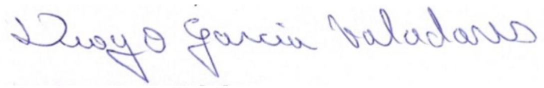

**Diogo.Valadares@cshs.org**
